# The mediation effect of vitamin A and vitamin D supplement in the association between serum vitamin K levels and musculoskeletal disorders in preschool children

**DOI:** 10.3389/fnut.2023.1239954

**Published:** 2023-12-22

**Authors:** Qiaoyue Ge, Lu Zhang, Zeyuan Sun, Jiarui Cai, Xia Jiang, Hong Wang, Xinxi Li, Chuan Yu, Chenghan Xiao, Zhenmi Liu

**Affiliations:** ^1^Department of Maternal and Child Health, West China School of Public Health and West China Fourth Hospital, Sichuan University, Chengdu, Sichuan, China; ^2^Institute of Systems Epidemiology, West China School of Public Health and West China Fourth Hospital, Sichuan University, Chengdu, Sichuan, China; ^3^Department of Child and Adolescent Psychiatry, School of Academic Psychiatry, Institute of Psychiatry, Psychology and Neuroscience, King's College London, London, United Kingdom; ^4^Department of Child Health Care of Sichuan Maternal and Child Health Hospital, Chengdu, Sichuan, China

**Keywords:** preschool children, serum vitamin K levels, musculoskeletal disorders, a cross-sectional study, vitamin A and vitamin D supplement

## Abstract

**Introduction:**

Vitamin K deficiency may elevate the incidence of musculoskeletal disorders (MSD), whereas it lacks validation for pediatric populations and has uncertain dose recommendations. In this context, we hypothesized that serum vitamin K levels are associated with MSD in preschool children, and the widely used vitamin A and vitamin D supplements may mediate these associations based on potential mechanisms, which expects to provide guidance for future practice.

**Methods:**

A cross-sectional study was conducted in Sichuan province in southwestern China, from January 2021 to May 2022. Serum levels of vitamin K1/K2 and 25(OH)D were determined using the high-performance liquid chromatography method, and the diagnosis of MSD was executed by clinicians. Overall and stratified logistic regression analysis based on categorized 25(OH)D levels were conducted to assess association between serum vitamin K levels and MSD prevalence after adjusting for confounders. Mediation analysis was further performed and vitamin A and D supplementation was regressed as the mediator.

**Results:**

A total of 6,368 children aged 0–6 years old were enrolled. MSD was identified in 1179 (18.51%) of the children, while 5,189 (81.49%) of them did not present such disorder. After adjusting confounders, a significant difference was found in serum vitamin K1 level between children in MSD and Non-MSD group (*OR* = 0.802, 95%*CI* 0.745–0.864). No significant difference was found in serum vitamin K2 level between the two groups (*OR* = 0.975, 95%*CI* 0.753–1.261). The association between vitamin K1 level and MSD prevalence was partly (36.8%) mediated by vitamin A and D supplementation.

**Conclusions:**

A low serum vitamin K1 level is connected with an increased risk of MSD among children, highlighting that vitamin A and D supplementation is a helpful intervention to prevent MSD in children with vitamin K deficiency.

## Background

Musculoskeletal disorders (MSDs) are conditions that can affect the muscles, bones, and joints, which are also the largest source of global rehabilitation need, ranking fifth in disability-adjusted life years (DALYs) and first in years lived with disability (YLDs) (1, 2). Childhood, an active stage of bone mineral accumulation and critical for adult peak bone mass (3, 4), is a particularly susceptible window for prevention and treatment of MSDs (5). MSDs in childhood, such as rickets, can lead to bone deformities and difficulties with activities, which impact health substantially, growth and development of children and adolescents (6, 7). Poor treatment of musculoskeletal problems can have a negative influence on the quality of life and lead to short-term consequences like fractures, or long-term ones like osteoporosis (8–12). This implies that MSDs in children should be given special attention due to their higher potential for skeleton healing and remodeling (5).

Age is not the sole factor influencing MSDs, as MSDs have been widely reported to be closely associated with nutrient intake: studies have shown that vitamin C can foster trabecular bone formation by modulating bone matrix gene expression (13, 14), while vitamin D and its metabolites contribute to bone development by affecting intestinal calcium transport and serum calcium and phosphate homeostasis (15, 16). Furthermore, vitamin A appears to affect bone health by simulating osteoblastic activity and bone formation, as well as inhibiting osteoclastic activity and bone resorption (17). It also correlates with the availability of vitamin D receptor binding vitamin D (18). Recent studies also suggest that vitamin K deficiency may serve as a predictor of MSDs by reducing the carboxylation of vitamin K-dependent proteins, such as osteocalcin and matrix Gla protein, which regulate the calcification (19–22), thereby paving the way for etiological investigations and interventions.

Some studies to date have examined the relationship between vitamin K and adult bone health, yielding some negative correlations between serum vitamin K levels and MSDs (23, 24). However, the study on vitamin K levels and its association with MSDs in children remains limited, particularly in preschool children who are experiencing active bone mineral accumulation. Vitamin K deficiency is one of the most common malnutrition issues in Chinese children (25). Therefore, it is urgent to explore the association between serum vitamin K levels in preschool children and their MSDs. In addition, due to the potential side effects of vitamin K (26), there is insufficient evidence to recommend vitamin K supplementation for children. However, some alternative interventions can be taken to reduce the adverse effects of vitamin K deficiency. For instance, vitamin A (Vit A) and vitamin D (Vit D) are easily accessible, safe, and widely prescribed in China (27–29), acting synergistically in bone metabolism with vitamin K. Extrapolating from general population studies (30, 31), it is plausible that Vit A and Vit D supplementation in MSD patients could serve as a potential therapeutic target to enhance bone health.

This study aimed to assess 1) the association of two major subtypes of vitamin K [phylloquinone (vitamin K1) and menaquinone (vitamin K2)] in a large Chinese sample of preschool children with MSD and 2) the mediation effect of Vitamin A/D supplement in the association between Vitamin K1/K2 levels and MSDs in preschool children.

## Methods

### Participants and data collection sample sources

**Figure 1:**
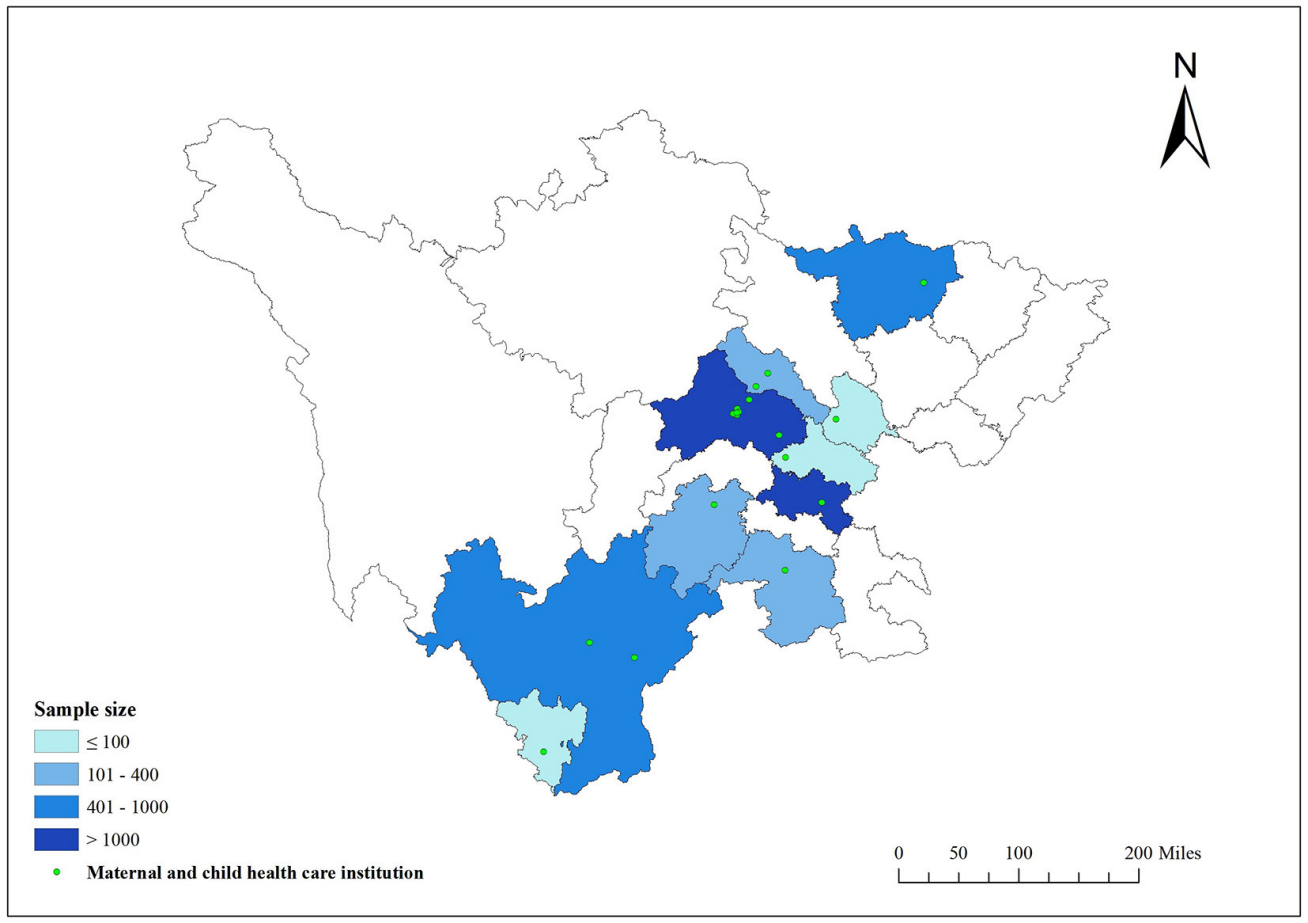
Distribution of 18 maternal and child health care institution in Sichuan province.

The study enrolled children aged 0–6 years old who underwent health assessments at outpatient clinics of the child health departments across 10 urban areas (32) comprising 18 maternal and child health hospitals in Sichuan Province (Figure 1) from January 2021 to May 2022. Variables considered in this study, including the participant's age, gender, parents' highest education, birth height, birth weight, gestational week and vitamin A and D supplementation reported by the participant's guardians at enrollment were extracted from medical records. Written informed consent was secured from their guardians before the study. Ethical approval for the study protocol was granted by the Ethics Committee of Sichuan Provincial Maternal and Child Health Care Hospital (2019, No. 20).

### Musculoskeletal disorders

In this study, children diagnosed with diseases such as O-leg, X-leg, pectus carinatum, square skull, and bone pain were classified as having an MSD. The diagnosis of these conditions was confirmed by clinicians according to the guidelines of Practical Child Health Care (33).

### Serum vitamin K1/K2 and 25(OH)D concentrations

The vitamin K and vitamin D levels in serum were assessed by high-performance liquid chromatography (HPLC) (34–36). Briefly, 2 ml of venous blood was collected in a standard biochemical tube (red cap tube) and stored at room temperature while being protected from light. Within 8 h of collection, the samples were tested. Following the internal labeling vitamin K with liquid-liquid extraction and isotope internal standard quantification, the internal standard working solution was transferred to a clear tube and subjected to centrifugation at 4,000 rpm for 10 min. The resulting supernatant was collected and blown dry before being mixed with methanol for 1 min. Lastly, vitamin K1, K2, as well as 25(OH)D in supernatant were quantified by HPLC (model L550).

### Covariates

Some relative covariates were considered in the analysis. Information about socio-demographic factors and birth information was obtained through the Pediatric Health Care System, which mainly contained: age (year), gender (male or female), residential area (ten urban areas in Sichuan province), birth height (meter), birth weight (kilogram), gestational week, and parent's degree (under junior high school, senior high school or technical secondary school and college and above).

### Statistical analyses

Categorical variables were reported in terms of numbers and percentages, while continuous variables were presented as means and standard deviations (SD). To compare the characteristics of MSD and NMSD groups, Pearson chi-square tests for categorical variables and *t*-test were employed for continuous variables. Missing values were identified and imputed by multivariate imputation using chained equations (37).

We constructed logistic regression models to examine the associations between levels of vitamin K1/K2 as well as 25(OH)D and MSDs, with the former treated as continuous variables. To further investigate the associations between vitamin K1/K2 level and MSDs conditional on 25(OH)D level, we conducted stratified analysis based on categorized 25(OH)D levels, with 25(OH)D level <12 ng/mL, ≤ 12- <20 ng/mL, and ≥20 ng/mL, representing normal, insufficient, and deficient levels of vitamin D, respectively (38). All models were adjusted for children's gender, residential area, birth height, birth weight, gestational week, and parents' highest education. Additionally, to assess the influence of serum vitamin K1/K2 concentrations on MSD via vitamin A and D supplementation, vitamin A and D supplementation were regressed on the mediator variables. In this pathway, direct, indirect, total effects and the proportion of mediation were estimated.

Furthermore, we conducted two sensitivity analyses to assess the robustness of our results. The first analysis excluded the missing data instead of using multivariate imputation, whereas the second analysis only included individuals with birth weight (2.5-4.0 kg) and gestational week (37-42 weeks) within the normal range.

All analysis were performed using R, version 4.0.3 (R Project for Statistical Computing). For all statistical tests, a significance level of two-tailed *P* ≤ 0.05 was employed. Estimated effects were presented with odds ratios (*OR*) and 95% confidence intervals (95%*CI*).

## Result

### Participant characteristics

Descriptive statistics regarding the sociodemographic characteristics of the survey sample are presented in Table 1 and Supplementary Table 2. In this study, all variables were described with numbers and percentages. A total of 6,368 children aged 0–6 years old were enrolled, with 1,179 (18.51%) identified having MSDs (subtypes of MSDs are shown in Supplementary Table 2). Notably, the MSD group exhibited a greater proportion of male children in comparison to the NMSD group (*P* < 0.001). Regarding age, the NMSD children averaged 2.84 years, whereas MSD children averaged 3.8 years, indicating that children of the NMSD group were significantly younger than their MSD counterparts (*P* < 0.001). Children in the MSD group exhibited greater birth weight, height and BMI than those in the NMSD group (*P* < 0.05). In terms of the parity, there was no significant difference between the two groups (*P* = 0.67). Our study revealed no significant gestational week disparities emerged between the groups (*P* = 0.06). However, compared with the MSD group, the NMSD group were more likely to receive vitamin A and D supplementation (50.99 vs. 31.30%, *P* < 0.001).

**Table 1:** Demographic characteristics for participants (*N* = 6,368).

**Exposure**	**Non-musculoskeletal disorders children**	**Musculoskeletal disorders children**	***P***
	***N =* 5,189 (81.49%)**	***N =* 1,179 (18.51%)**	
**Gender**			<0.001
Male	2,914 (56.16%)	726 (61.58%)	
Female	2,275 (43.84%)	453 (38.42%)	
**Age**	2.84 ± 1.59	3.80 ± 1.58	<0.001
**Gestational week**	38.62 ± 1.46	38.72 ± 1.63	0.06
**Parity**			0.662
1	3,484 (67.14%)	798 (67.68%)	
2	1,562 (30.10%)	354 (30.03%)	
≥3	143 (2.76%)	27 (2.29%)	
**Birth weight**	3.15 ± 0.42	3.20 ± 0.41	<0.001
**Birth height**	49.64 ± 1.30	49.77 ± 1.24	<0.001
**Birth BMI**	12.75 ± 1.39	12.88 ± 1.39	0.003
**Current BMI**	16.00 ± 16.82	15.69 ± 11.14	0.455
**Vitamin AD supplementation**			<0.001
No	2,543 (49.01%)	810 (68.70%)	
Yes	2,646 (50.99%)	369 (31.30%)	

Data are mean ± SD or n (%).

### Serum vitamin K1/K2 concentrations for children

**Figure 2:**
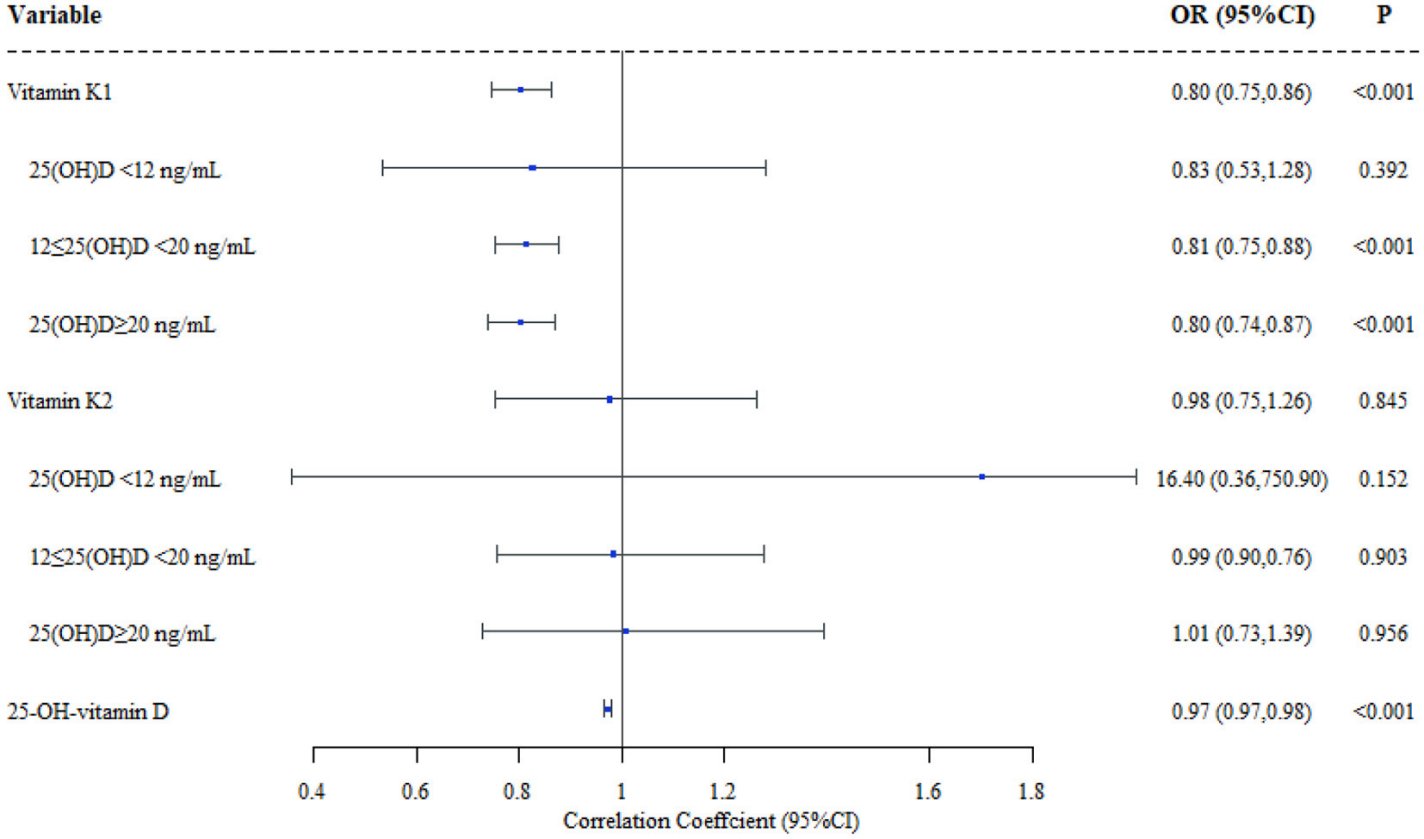
Effect of vitamin K1/K2/25(OH)D in children's musculoskeletal disorders (*N* = 6368). Adjustment of area, gender, birth height, birth weight, gestational week and parents' highest education.

Logistic regression models were applied to investigate the relationship between serum vitamin K1/K2 concentrations and the status of MSD in children. The models were adjusted for confounders, including residential area, kid's gender, birth height, birth weight, gestational week and parents' highest education. Our results revealed a significant difference in serum vitamin K1 levels between children in MSD and NMSD groups (*OR* = 0.802, 95%CI 0.745–0.864), however, there was no difference in serum vitamin K2 levels between the two groups (*OR* = 0.975, 95%CI 0.753–1.261). To further examine whether 25(OH)D levels modified the associations, we constructed stratified logistic regression models. The results indicated that there was no difference in the vitamin K1/K2 level among MSD and NMSD children in the vitamin D deficiency group (<12 ng/mL). Moreover, when the concentration of 25(OH)D in the serum increases as 12 ng/mL ≤ 25(OH)D <20 ng/mL or ≥20 ng/mL, vitamin K1 level was significantly associated with MSDs (95%*CI* 0.754–0.879,95%*CI* 0.740–0.871), while the vitamin K2 level remained similarly non-significant (95%*CI* 0.758–1.277,95%*CI* 0.730–1.394). More details are shown in Figure 2 and Supplementary Table 3.

### Mediation of vitamin A and D supplementation

A mediation of the association between serum vitamin K1 and MSD through the vitamin A and D supplementation mediator was found. The total effect was highly significant (β = −0.024 *P* < 0.001). Likewise, the direct and indirect effects were β = −0.015 (*P* < 0.001) and β = −0.008 (*P* < 0.001), respectively. The proportion of the effect of vitamin K1 levels on the MSD that goes through vitamin AD supplementation is 36.8%. Conversely, the difference in the mediation of the association between serum vitamin K2 levels and MSD was not statistically significant. A comprehensive display of the total, direct, indirect effects and the proportion of mediation are displayed in Table 2.

**Table 2:** The result of mediation analysis from vitamin K to MSD via vitamin AD supplementation.

**Variable**		**Estimate**	**95% confidence**		***P***
			**Lower**	**Upper**	
Vitamin K1	ACME	−0.008	−0.012	−0.010	<0.001
	ADE	−0.015	−0.022	−0.010	<0.001
	Total effect	−0.024	−0.032	−0.020	<0.001
	Prop. mediated	0.368	0.267	0.520	<0.001
Vitamin K2	ACME	−0.006	−0.016	0.000	0.038
	ADE	0.001	−0.032	0.050	0.988
	Total effect	−0.005	−0.039	0.040	0.728
	Prop. mediated	1.297	−3.793	7.550	0.738

ACME, stands for average causal mediation effects; ADE, stands for average direct effects; Total Effect, stands for the total effect (direct + indirect) of the VK1 on the MSD; Prop. Mediated, describes the proportion of the effect of the VK1 on the MSD that goes through the mediator (Vitamin AD supplementation).

### Sensitive analysis

Sensitivity analyses employing logistic regression models with missing values removed (Table 3, model 1) yielded similar results, indicating a negative correlation between serum vitamin K1 concentrations and MSD prevalence (*OR* = 0.810, 95%*CI* 0.749–0.876), and no significant correlation for serum vitamin K2 concentrations (*OR* = 0.878, 95%*CI* 0.648–1.190). Furthermore, the results of logistic regression models involving participants with normal birth weight (2.5–4.0 kg) and gestational week (37-42 weeks) were consistent with the main findings (Table 3, model 2).

**Table 3:** Sensitive analysis—effect of vitamin K1/K2 in musculoskeletal disorders.

**Model**	**Exposure**	**OR**	**95% confidence**		***P***
			**Lower**	**Upper**	
Model 1	Vitamin K1	0.810	0.749	0.876	<0.001
	Vitamin K2	0.878	0.648	1.190	0.401
Model 2	Vitamin K1	0.818	0.757	0.883	<0.001
	Vitamin K2	0.912	0.671	1.239	0.554

Adjustment of age, area, gender, birth height, birth weight and gestational week.

Model 1 excluded the missing data instead of using multivariate imputation.

Model 2 only included individuals with birth weight (2.5-4.0 kg) and gestational week (37-42 weeks) within the normal range.

## Discussion

The study examined the association between serum vitamin K concentration and MSDs among preschool children in Sichuan, China. Our findings demonstrate a significant association between serum vitamin K1 level and MSDs, after adjusting for children's gender, residential area, parents' highest education, birth height, birth weight and gestational week at birth. This association attenuated to null only when 25(OH)D was below <12 ng/ml. Moreover, vitamin A and D supplementation may serve as a potential influence on these associations among children. However, the serum vitamin K2 level was not associated with MSDs in any models.

To the best of our knowledge, limited studies have addressed the association between vitamin K and MSDs in preschool children (39–42). Our data indicate a significant association between serum vitamin K1 level and MSDs in children aged 0-6 years, highlighting the importance of addressing vitamin K1 deficiency and fostering proper nutrient supplementation and dietary habits in this population. A study also revealed that vitamin K1 had a greater effect on the incidence of bone fractures compared to vitamin K2 (43) which yielded results consistent with ours in general. However, findings regarding the efficacy of Vit K supplementation on bone are yet inconclusive (44, 45). While a meta-analysis has reported modest overall treatment effects (46), others have found no significant effect (47, 48). Furthermore, no randomized controlled trials have evaluated the effects of comparable doses of various forms of vitamin K on skeletal outcomes (49). Two Cochrane reviews have shown that the dosage and safety of vitamin A and D supplementation in children has been well established (50, 51). In China, vitamin A and D supplementation follows a standard, strict, and safe implementation protocol (52). Since 2013, the Chinese market for vitamin A and D drops or tablets has grown rapidly and it is widely utilized in Chinese children. Based on this study, vitamin A and D supplementation is an effective and feasible measure for children to partially block skeletal muscle disease caused by vitamin K deficiency.

Though the mechanisms linking nutrients deficiencies and bone metabolism are not fully understood, previous studies show that vitamin K may influence bone transformation through various pathways, including osteoblast differentiation, osteoclast inhibition, and activation of bone-associated vitamin K-dependent proteins, such as osteocalcin and matrix Gla protein that play essential roles in the mineralization of extracellular bone matrix (43, 53, 54). During this process, bone mineralization (55) is also closely related to the serum calcium levels, which can be regulated by vitamin D (56–58). This is consistent with our findings. It could be due to excessively low levels of vitamin D, incomplete decarboxylation of osteocalcin cannot occur which leads to inhibited bone mineralization, further manifesting as the lack of correlation between level of vitamin K and MSD occurrence. Besides, vitamin A also appears to affect bone health by simulating osteoblastic activity and inhibiting osteoclastic activity (18). Just as vitamin A and vitamin D are often used together in clinical practice, evidence shows that their pathways may overlap (59). Based on the above knowledge, we can speculate that supplementation with vitamin A or vitamin D or a combination may be a potential mediator to mitigate the adverse effects of vitamin K deficiency on MSDs, which is consistent with our study.

Our findings provide several implications for future research future research and practice. More attention should be paid to vitamin K deficiency in children, and vitamin A and D supplementation may help mitigate the adverse effects of vitamin K deficiency on MSDs. What's more, large prospective cohorts should examine the short- and long-term effects of vitamin K level on bone health in children. Ethically feasible clinical trials with safe doses can be conducted to provide further insights.

There are some strengths in this study. Firstly, we used high-performance liquid chromatography (HPLC) for vitamin K levels determination of participant from multiple regions with guaranteed consistency of measurement methods and a high detection sensitivity. Secondly, diagnosis of MSD was confirmed through physicians at the Sichuan Maternal and Child Health Hospital. The recollection bias and reporting bias has been controlled. This study is also restrained by several limitations. Firstly, the present study is a cross-sectional study that can merely show the correlation instead of causality. Secondly, despite careful adjustment for a extent range of covariates in the model, residual confounding due to the unavailability of data may exist. Thirdly, we only detected serum level of menaquinone-4, one of the subtypes of vitamin K2, which may underestimate level of exposure. Lastly, our study is conducted in a regional population with specific age group. We recommend exercising caution when extrapolating the findings of this study to children in other regions and age groups.

## Conclusions

This study found that the serum vitamin K1 levels was significantly associated with MSDs, while the serum vitamin K2 levels were not. It also illustrated that vitamin AD supplementation may potentially influence these associations. These findings underscore the public health significance of vitamin A and D supplementation as a beneficial intervention for preventing MSDs attributable to vitamin K1 deficiency in children.

## Data availability statement

The raw data supporting the conclusions of this article will be made available by the authors, without undue reservation.

## Ethics statement

The studies involving humans were approved by Ethics Committee of Sichuan Provincial Maternal and Child Health Care Hospital (2019, No. 20). The studies were conducted in accordance with the local legislation and institutional requirements. Written informed consent for participation in this study was provided by the participants' legal guardians/next of kin.

## Author contributions

QG and LZ are the main researchers, who has done the major work of data analysis and collection, respectively. ZS and JC are two of the main members of the supporting team, supervised by ZL, which plays an active role from the design of the study to the final drafting. The team also consists of other co-authors, including XJ, HW, XL, CY, and CX. Together, the study was guided by ZL throughout the process. All authors contributed to the article and approved the submitted version.
