# Supplementary material for: The mediation effect of vitamin A and vitamin D supplement in the association between serum vitamin K levels and musculoskeletal disorders in preschool children
Source: Front Nutr. 2023 Dec 22;10:1239954. doi: 10.3389/fnut.2023.1239954 (PMC10766770; doi:10.3389/fnut.2023.1239954)
Supplement: Supplementary file 1 [file Data_Sheet_1.docx]

| **Exposure** | **Non-Musculoskeletal disorders children** | **Musculoskeletal disorders children** | | ***P*** |
| --- | --- | --- | --- | --- |
|  | **N=5189 (81.49%)** | | **N=1179 (18.51%)** |  |
| **Parents' highest education** |  | |  | 0.006 |
| Under junior high school | 1381 (26.61%) | | 346 (29.35%) |  |
| Senior high school or technical secondary school | 3052 (58.82%) | | 634 (53.77%) |  |
| College and above | 756 (14.57%) | | 199 (16.88%) |  |

**Supplementary Table 1**: The distribution of parents’ highest education level (N=6368)

Data are mean ± SD or n (%)

**Supplementary Table 2.** The distribution of MSDs subtypes (N=1179).

| **Subtypes** | **n (%)** |
| --- | --- |
| **O-leg** | 33 (2.80%) |
| **X-leg** | 159 (13.49%) |
| **Pectus carinatum** | 124 (10.52%) |
| **Square skull** | 4 (0.34%) |
| **Bone pain** | 859 (72.85%) |

**Supplementary Table 3.** Effect of Vitamin K1/K2/25(OH)D in Children’s Musculoskeletal Disorders (N = 6368)

| **Variable** | **OR** | **95% confidence** | | ***P*** |
| --- | --- | --- | --- | --- |
|  |  | **lower** | **upper** |  |
| **Vitamin K1** | 0.802 | 0.745 | 0.864 | <0.001 |
| 25(OH)D <12 ng/mL | 0.826 | 0.534 | 1.279 | 0.392 |
| 12≤25(OH)D <20 ng/mL | 0.814 | 0.754 | 0.879 | <0.001 |
| 25(OH)D≥20 ng/mL | 0.803 | 0.740 | 0.871 | <0.001 |
| **Vitamin K2** | 0.975 | 0.753 | 1.261 | 0.845 |
| 25(OH)D <12 ng/mL | 16.396 | 0.358 | 750.891 | 0.152 |
| 12≤25(OH)D <20 ng/mL | 0.984 | 0.758 | 1.277 | 0.903 |
| 25(OH)D≥20 ng/mL | 1.009 | 0.730 | 1.394 | 0.956 |
| **25-OH-vitamin D** | 0.972 | 0.966 | 0.979 | <0.001 |

Adjustment of area, gender, birth height, birth weight, gestational week and parents' highest education
